# Wettability of HPMC/PEG/CS Thermosensitive Porous Hydrogels

**DOI:** 10.3390/gels9080667

**Published:** 2023-08-18

**Authors:** Li Ma, Tong Shi, Zhaoyun Zhang, Xixi Liu, Hui Wang

**Affiliations:** 1 College of Safety Science and Engineering, Xi’an University of Science and Technology, Xi’an 710054, China; 2 Jinduicheng Molybdenum Industry Co., Ltd., Xi’an 710077, China; 3 Xinlong Coal Mining, Yanzhou Coal Mining Energy Group Co., Ltd., Zoucheng 273513, China

**Keywords:** thermosensitivity, porous hydrogel, spreadability, adhesivity, wettability

## Abstract

Thermosensitive hydrogels have been receiving attention in the development of fire extinguishing agents due to their stimuli responsivity. Conventional hydrogels are limited by their slow response rate, and their wettability has not been studied systematically. In the present study, a concentrate of a thermosensitive porous system has been successfully synthesized by adding Na_2_CO_3_/CH_3_COOH as a foaming agent into the mixture of hydroxypropyl methylcellulose (HPMC)/polyethylene glycol (PEG)/chitosan (CS). The systems with different concentrations were obtained by diluting the concentrate with water. Thermosensitivity, surface tension and contact angle were characterized. In addition, spreadability, wettability and adhesivity were investigated systematically. Results showed that the systems with a concentration greater than 15 wt% exhibited outstanding performance of thermosensitivity and coagulability. A total of 20 wt% of the system has the best spreadability and wettability on the wood surface, most likely due to favorable contributions brought by both adequate viscosity and hydrophilicity. The adhesive force and surface-free energy of the pre-gel droplet that reached deposition on the wood surface decreased by 46.78% and 20.71%, respectively. The gel has a great capacity of water retention over a long period of time, which makes this porous gel the best system when it comes to its wettability and adhesiveness towards the chosen wood surface. The equilibrium surface tension decreased by 45.50% compared with water. HPMC/PEG/CS thermosensitive porous hydrogel with excellent wettability presented wide-ranging possibilities for the further development of fire suppression agents of fast phase-transition thermosensitive hydrogel.

## 1. Introduction

Water is the most commonly used fire suppression agent for Class A. The fire extinguishing performance of water can be enhanced by improving its wettability and adhesion after adding additives to water, such as emulsifiers, thickeners and surfactants [1,2,3]. Gels used as fire suppressants have been developed because they integrated the advantages of good water retention, adhesion and plugging. Most firefighting gels showed poor wettability on combustibles as a consequence of high viscosity [4,5]. Thermosensitive hydrogels have recently been receiving increased attention in many industrial fields due to their unique stimuli-responsivity characteristics [6,7,8]. They exhibit excellent fluidity and wettability at room temperature and are transformed into gels when the temperature reaches a lower critical solution temperature (LCST) [9]. Developing a new type of water-based extinguishing agent using thermosensitive hydrogels is a promising approach.

Natural hydrogels, such as cellulose and chitosan (CS), have the advantages of wide-ranging sources and excellent thermosensitivity [10]. Hydroxypropyl methylcellulose (HPMC) is a typical non-ionic cellulose ether with great thermosensitivity, water retention and adhesion thickening [11,12,13]. CS is a biodegradable amino polysaccharide. They are similar in structure and have high compatibility. HPMC/CS thermosensitive hydrogels can be constructed by the formation of intermolecular hydrogen bonds between the hydrophilic moieties of the macromolecular, chains of HPMC (hydroxyl groups), CS (amine, amide, hydroxyl groups) and water [14]. Li [15] et al., Wang [16] et al. and Hu [17] et al. synthesized HPMC/CS hydrogels with outstanding thermosensitivity and minimal cytotoxicity through physical cross-linking. The hydrogels could be fully gelled within 900 s above LCST [16]. Nevertheless, HPMC/CS hydrogels have been limited for long phase-transition response time and unstable gel-shrinkage structure after dehydration. Poly (ethylene glycol) (PEG) is a hydrophilic polymer containing a large number of ethyleneoxy units, which can improve the hydrophilicity and rheological property of hydrogels by forming physical cross-linking with an HPMC/CS mixture [18,19].

The current research has shown that changes in the wettability of the thermosensitive hydrogels are due to the phase transformation behavior. The wettability of smart materials triggered by a small change in temperature resulting in altering their hydrophilic–hydrophobic properties is required [20]. Ma et al. [21] found that the surface tension of a methylcellulose/sodium polyacrylate/magnesium chloride decreased after phase transition, enhancing its capability to wet the wood and the fire extinguishing efficiency. Gels with porous structure can promote not only swelling capacity but also water-retaining capacity and mechanical strength [22,23,24]. Zhang et al. [25] proved that the polyHIPE macro-porous hydrogels exhibit rapid swelling and good cooling performance. Xiao et al. [26] and Yang et al. [27] concluded that poly (N-isopropyl acrylamide-co-sodium acrylate) thermosensitive porous hydrogels exhibit excellent liquidity at low temperatures, thereby forming gels at temperatures above LCST, with high viscosity, strong adhesion and great water retention. They could be considered effective barriers to isolating the supply of oxygen from the surface of combustibles. An in-depth study of the wettability of thermosensitive hydrogels, especially the wetting process of hydrogels during the sol-gel process, has not been conducted.

HPMC/PEG/CS mixtures have great thermosensitivity and hydrophilicity [28]. In the present study, thermosensitive porous hydrogels were prepared based on the mixture of HPMC/PEG/CS (HPC porous hydrogels) and sodium carbonate/acetic acid (Na_2_CO_3_/CH_3_COOH) as the foaming agent. The spreadability, wettability and adhesivity of the hydrogels were investigated systematically during phase transition. The results can provide a theoretical foundation for the application of fast phase-transition thermosensitive porous hydrogels in the development of a new type of water fire-extinguishing agents.

## 2. Results and Discussion

Table 1 shows the formulations of the HPC porous hydrogels with different concentrations obtained by diluting the initial concentrate with water. The LCST values of all samples were 67 °C. The weight ratio between the macromolecular components (HPMC; PEGCS) into the mixed HPC systems were 3 wt%:1.5 wt%:0.8 wt%.

### 2.1. Basic Properties of the HPC Porous Systems

**Figure 1:**
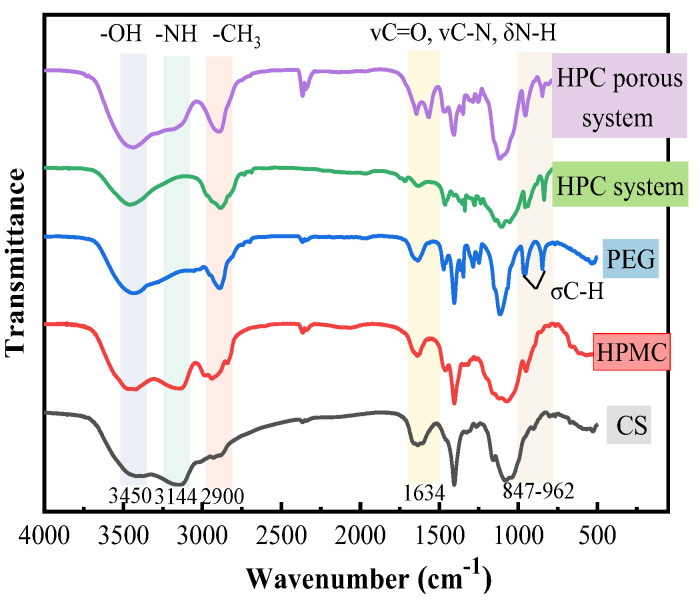
FT-IR spectra of the HPC porous system and HPC system.

Figure 1 shows the FT-IR spectra of the HPC porous system, the HPC system, PEG, HPMC and CS. In the HPC porous system and the HPC system, the stretching vibration absorption peak of the hydroxyl group appeared at 3441 cm^−1^, and the –NH_2_ peak was observed within the range of 3130–3230 cm^−1^. The area of –OH and –NH peaks significantly decreased compared with HPMC and CS. This result showed that –OH and –NH_2_ formed intermolecular hydrogen bonds with water molecules having different strengths [16]. The area of –CH_3_ peak in the HPC porous system and HPC system at 2900 cm^−1^ increased and had a blue shift compared with HPMC and PEG, which was caused by the superposition of –CH_2_CH_2_ in HPMC and –CH_3_ in PEG. The superposition of the CS characteristic peaks of the remaining N-acetyl groups (C=O and C–N stretching, N–H bending) is observed at 1634 cm^−1^. The PEG characteristic peak σC–H and out-of-plane bending vibration peaks were observed at 847–962 cm^−1^. The characteristic peaks and hydrogen bonds between molecules in the HPC porous system were observed, indicating that the system was successfully prepared by physical cross-linking.

**Figure 2:**
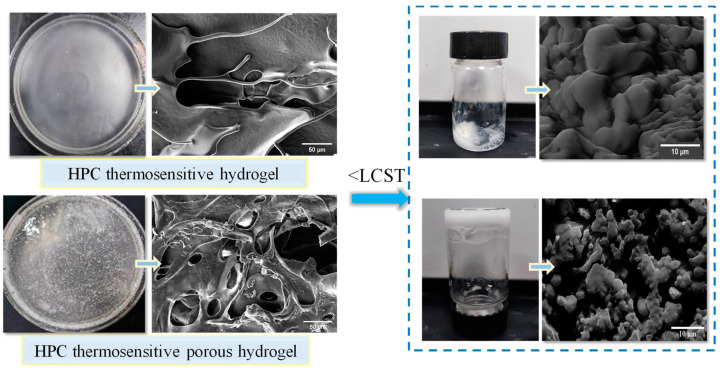
Morphology of the HPC systems concentrates during phase transition.

The LCST of the undiluted HPC porous system concentrate was 67 °C, and the phase-transition response time at this point was 28 s. The phase transition response rate of the HPC porous hydrogel concentrate was significantly faster than that of the CS/HPMC/glycerol solutions and HPC thermosensitive hydrogel [16,28]. The 500× magnification of pre-gel morphology below LCST and the gel framework at 8000× magnification above LCST for undiluted HPC porous system compared with the corresponding non-porous ones are shown in Figure 2. The undiluted HPC porous system (bottom side, Figure 2) displayed many more micropores, mesopores and macropores compared with the HPC system (upper side, Figure 2) when the temperature was lower than LCST, due to the CO_2_ gas generated by the chemical reaction between CH_3_COOH and Na_2_CO_3_. In addition, the system phase has changed into a gel one at temperatures above LCST. The desorption of water molecules (bound water to free water) from the ether moieties (belonging to methoxy and ethyleneoxy groups of HPMC and PEG, respectively) and then by intermolecular aggregation of these hydrophobic regions eventually lead to a 3D polymer network with bulk water entrapped inside it (hydrogel physically crosslinked) [29,30]. The surface of the HPC thermosensitive hydrogel (upper right, Figure 2) concentrate folded into a worm-like 3D structure at 8000× magnification after phase transformation. This effect should be ascribed to the action of the hydrophobic domain that promoted the shrinkage of molecular chains and the discharge of free water. The gel after the phase transition of the HPC porous system concentrate has an interpenetrating, unbroken and porous framework with low filling density. The finding revealed that the unbroken and interpenetrating 3D network structure could be formed by the formation of carbon dioxide by the foaming agent physical cross-linking between HPMC, CS and PEG [16,19]. In addition, the porous structure could maintain a stable structure under heat action and avoid the collapse of the framework [28].

**Figure 3:**
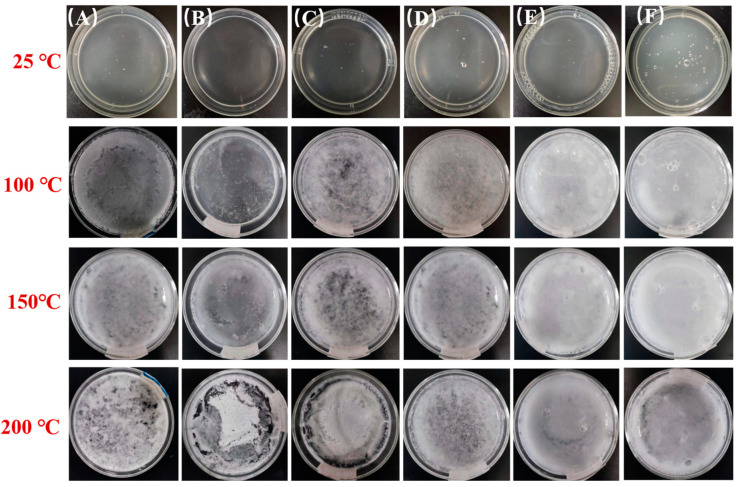
Morphology of the HPC porous systems was maintained at 600 s with different concentrations of 25 °C, 100 °C, 150 °C and 200 °C: (**A**) P-0; (**B**) P-5%; (**C**) P-10%; (**D**) P-15%; (**E**) P-20%; (**F**) P-25%.

Figure 3 shows that the morphology of the HPC porous systems was maintained at 600 s with different concentrations and temperatures.

As shown in Figure 3, the HPC porous systems displayed an appearance of uniform dispersions with good fluidity at 25 °C and a gel consistency at temperatures above LCST with advanced coagulability at elevated temperatures. The HPC porous systems above their sol-to-gel transition temperature exhibited a heterogeneous aspect when the overall concentration of the macromolecular components was lower than or equal to 15%. These systems became films of dry gels with a visible non-homogeneous morphology and poor wettability due to the rapid water evaporation at 200 °C. This behavior was not observed at lower temperatures, as shown in Figure 3. This behavior could be attributed to the polymer chains shrinkage after losing water on continuous heating, which eventually damaged the uniform structure of the gel network. Outstandingly, the HPC porous systems with a concentration greater than 15 wt% exhibited an almost intact wet gel film at 200 °C. The water retention and thermal stability of gels were significantly improved given that the porous structure immobilized more bound water strongly linked to the polymer chains of the macromolecular network most likely through hydrogen bonding [28].

**Figure 4:**
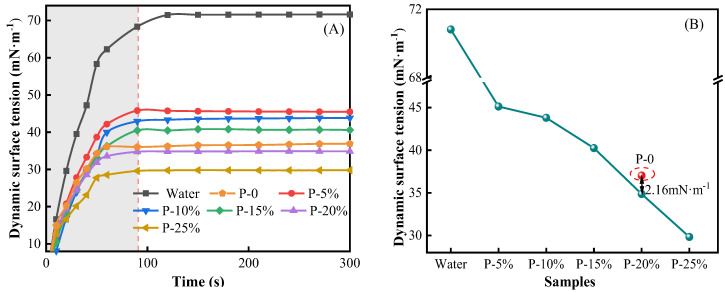
(**A**) The dynamic surface tension of the HPC porous systems at 25 °C. (**B**) The equilibrium surface tension of the HPC porous systems at 25 °C.

Figure 4 shows the dynamic surface tension and the equilibrium surface tension of the HPC porous systems with different concentrations at 25 °C.

It can be seen in Figure 4 that the dynamic surface tension curves of all systems increased rapidly and then flattened gradually over time. The dynamic surface tension of water tended to balance at 120 s, with an equilibrium value of 70.82 mN/m. The dynamic surface tension of all HPC porous systems tended to reach the equilibrium values after 90 s. This result was mainly due to attaining constant, equilibrium cohesive interactions between chemical entities contained in the thin layer located at the surface of the systems. The hydrogen bonds web associated with water molecules distributed within superficial layer (at water–air interface) led to such a high value of surface tension (or liquid air interfacial tension) for pure water. On the other hand, a certain, non-negligible surface-active character of the macromolecular compounds in the studied aqueous HPC systems can cause a progressive decrease of surface tension with elevating polymer concentration [31,32]. Furthermore, the surface tension of P-20% is 2.16 mN/m lower than that of P-0 (Figure 4B displayed) because the sodium acetate in the foaming agent ionized hydrophilic acetate ion (CH_3_COO^−^), further promoted the formation of hydrogen bonds with water and further enhanced the hydrophilicity [33]. The equilibrium surface tension of P-20% decreased by 20.69% compared with that of the P (NIPA-co-SA) thermosensitive hydrogel [34].

### 2.2. Spreadability of the HPC Porous Systems on the Wood Surface

#### 2.2.1. Spreading Behavior of the Aqueous Pre-Gel Droplets

**Figure 5:**
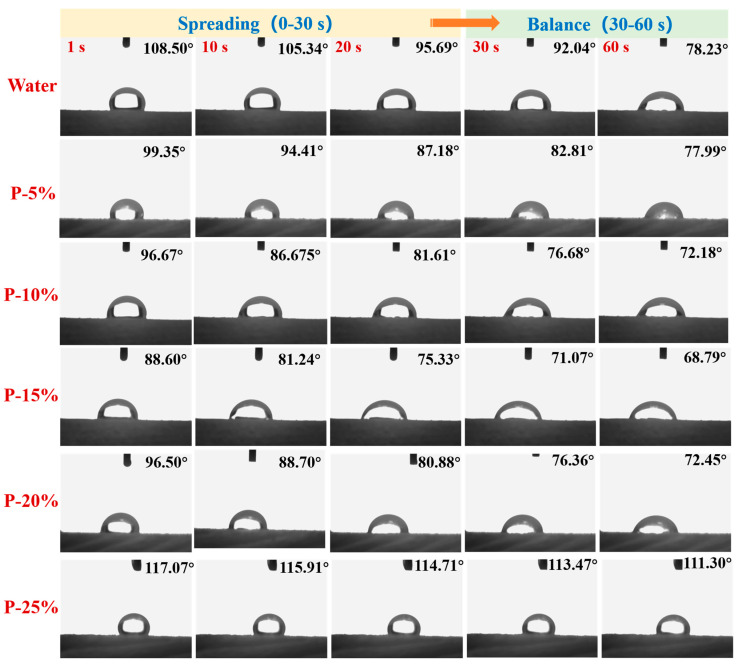
The spreading tendency of the HPC porous pre-gel systems with different concentrations on the wood surface.

The spreading tendency of the investigated HPC porous pre-gel systems based on the values of contact angles measured for the corresponding droplets as a function of time. The spreading tendency of the HPC porous pre-gel systems with different concentrations on the wood surface is shown in Figure 5.

Figure 5 showed that the contact angle of water dropped more evidently than the pre-gel droplets within 60 s. Water cannot easily spread and adhere to the wood surface due to its large surface tension and low viscosity. The range width of the contact angle values for HPC porous pre-gel droplets decreased gradually within 30–60 s with the increase in concentrations. The result demonstrated that pre-gel droplets spread continuously and reached the state of deposition on the hydrophilic wood surface. Actually, the droplet adsorption on the hydrophilic wood surface (as a time-dependent process) gradually increased because pre-gel droplets have good hydrophilicity. Nevertheless, the inertia force and the hydrophilic character of the liquid-solid interface greatly influenced the spreading behavior of droplets. Thus, P-25% adhered on the wood surface without spreading after dripping. This could be ascribed to the high viscosity of the droplets that significantly delayed the spreading process and made the inertial contribution negligible. According to the results of spreading behavior, P-15% and P-20% exhibited the most desirable characteristics of hydrophilicity and spreadability on the wood surface.

**Figure 6:**
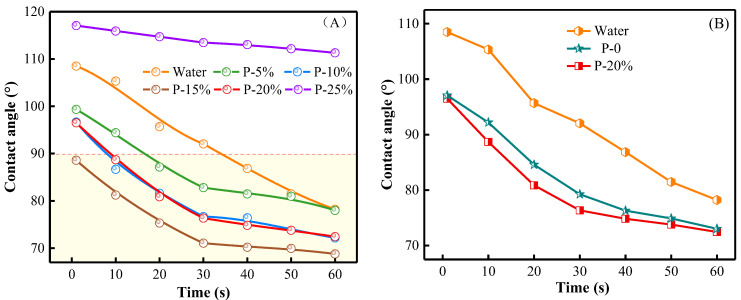
(**A**) Time evolution of the contact angle for the investigated HPC porous systems. (**B**) Variation of the contact angle for water, P-0 and P-20% within 60 s.

Figure 6A shows the contact angle of the HPC porous systems with different concentrations deposited on the wood surface versus time. Figure 6B displays variation in the contact angle of water, P-0 and P-20% within 60 s.

As shown in Figure 6, the contact angle decreased gradually with increasing the polymer content of the specified aqueous systems when the concentration was equal to or less than 15 wt%. This is mainly due to the presence of HPMC, CS and PEG bearing a large number of hydroxyl groups, which in turn led to a very favorable interaction with the hydrophilic wood substrate and, consequently, to a smaller and smaller contact angle as polymer content progressively rose. When the concentration of the HPC porous systems was higher than or equal to 20%, the values of contact angles significantly increased most likely due to the elevated values of the overall viscosity, which kept the nearly spherical shape of the pre-gel droplets unchanged after their deposition onto the wood surface. On the other hand, all the systems exhibited decreasing time-dependencies of contact angle, with a course gradually tending to equilibrium values. Practically, the spreading ability of these aqueous systems on the same wood substrate enhances as the values of contact angle decline. In addition, P-0 and P-20% showed similar magnitude and tendency of the time evolution of contact angle, which indicates that the foaming agent played an almost negligible role in altering the spreading properties of the specified systems.

#### 2.2.2. Spreading Coefficient of the Aqueous Pre-Gel Systems

To quantitatively analyze the spreadability, the spreading coefficient *S* for water, P-0, P-15%, and P-20% on the wood surface was calculated according to Equation (1) as follows [35]:

(1) $$S=\gamma_{LV}\left(\cos\theta_{t}-1\right)$$

where $\gamma_{LV}$ is the equilibrium surface tension of gas–liquid interface (in mN·m^−1^) and $\theta_{t}$ is the contact angle at time *t* (in °).

**Figure 7:**
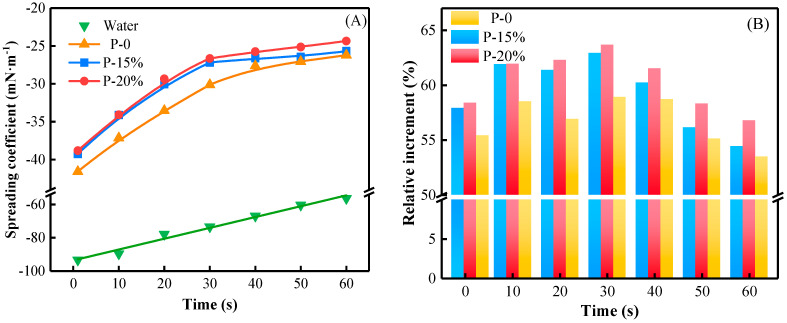
(**A**) Spreading coefficient of the investigated pre-gel droplets within 60 s. (**B**) Relative increment compared with water of spreading coefficient of P-0, P-15% and P-20%.

The closer *S* is to 0, the closer the droplet is to complete spreading. The spreading coefficients of water, P-0, P-15% and P-20% pre-gel droplets within 60 s are shown in Figure 7A. Figure 7B displays the relative increase (expressed in %) in spreading coefficients (relative increments) for the pre-gel systems with respect to those for water. It can be observed that the spreading coefficients for the pre-gel droplets increased exponentially with time, while the spreading coefficient of water increased linearly. These results are in accordance with the large values of contact angle for water (on the wood surface) mostly due to its high surface tension, which made the process of spreading on the specified substrate very difficult. The spreading coefficient of P-20% was closer to 0 compared to the relative increase (5.18 and 7.10%, respectively) in spreading coefficients (relative increments) for the pre-gel systems with respect to those for water and the other two chosen pre-gel systems, P-15% and P-0. Thus, its values (−38.82, −26.36 and −24.36 mN/m at 0, 30 and 60 s, respectively) were higher than those for P-0 by 6.64, 13.10 and 7.64% considered at the same time values. Due to a lower surface tension associated with a smaller contact angle measured onto the wood surface, P-20% was considered the most suitable from a spreadability and hydrophilicity standpoint in terms of its interaction with the chosen solid substrate.

### 2.3. Adhesivity of the HPC Porous Hydrogels on the Wood Surface

#### 2.3.1. The Adhesive Force of the Aqueous Pre-Gel Droplets

The minimum force required for the liquid to separate from the solid surface is defined as the adhesive force *W_a_*. It is calculated according to Equation (2) as follows [36]:

(2) $$W_{a}=\gamma_{SV}+\gamma_{LV}+\gamma_{SL}$$

By considering the Young’s equation, relationship (2) is transformed into the following:

(3) $$W_{a}=\gamma_{LV}\left(\cos\theta_{t}+1\right)$$

where $\gamma_{SV}$ is the equilibrium surface tension of the liquid–solid interface (in mN·m^−1^) and $\gamma_{SL}$ is the equilibrium surface tension of the gas–solid interface (in mN·m^−1^).

**Figure 8:**
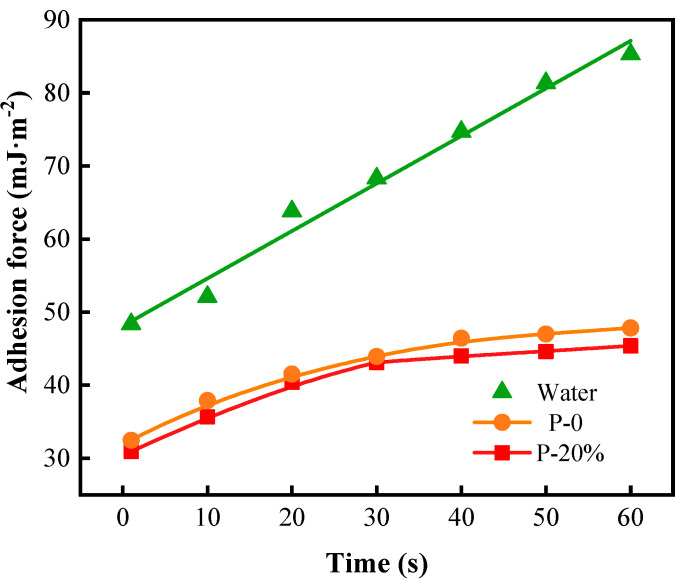
The adhesive force of water, P-0 and P-20% pre-gel droplets on the wood surface within 60 s.

Figure 8 shows the adhesive force of water, P-0 and P-20% pre-gel droplets. The adhesive force and the spreading coefficient of water, P-0 and P-20% droplets evolve similarly in time. Water could not reach values of adhesive force close to the equilibrium ones due to a gradual increase in water adsorption onto the hydrophilic wood surface. Instead, after a stage of the higher rate of initial rising in adhesive force, both P-0 and P-20% showed almost equilibrium values of adhesive force within the time range of 40–60 s when the most stable state of deposition has been practically attained. The adhesive force of P-0 and P-20% decreased by 43.91% and 46.78%, respectively, by comparison with that for water at 60 s most likely due to a lower molecular polarity of the pre-gel droplets at the liquid–solid (wood surface) interface, with a direct consequence in weakening the adhesiveness of these pre-gel systems onto the wood substrate. These data recommend P-20% as an optimal system regarding spreadability on the chosen solid substrate.

#### 2.3.2. Adhesivity of the Gels after the Phase Transition

The adhesion weight ratio of water, P-0 and P-20% of gels after phase transition are shown in Table 2. The experimental data indicated an almost negligible adhesivity of water toward the wood surface used at 300 and 600 s. The water on the wood surface was lost rapidly due to its high fluidity and large contact angle and rate of evaporation. These data recommend P-20% as an optimal system in terms of adhesivity on the selected solid substrate. It could be considered an effective barrier to isolate the supply of oxygen from the surface of the wood.

### 2.4. Wettability of the HPC Systems

#### 2.4.1. The Surface-Free Energy of the Wood Surface Wetted by the Aqueous Pre-Gel Droplets

The change of the surface-free energy of a solid surface (wood in this case) wetted by a liquid deposition on it (particularly, water and pre-gel droplets) can quantitatively characterize the phenomena of liquid adsorption and wettability and is defined according to Equation (4) proposed by Extrand as follows:

(4) $$\Delta G=\frac{RT}{3}\ln\left[\frac{\left(1-\cos\theta_{t}{)}^{2}(2+\cos\theta_{t}\right)}{4}\right]$$

where *R* is the gas constant (in J·(mol·K)^−1^) and *T* is ambient temperature (in K).

**Figure 9:**
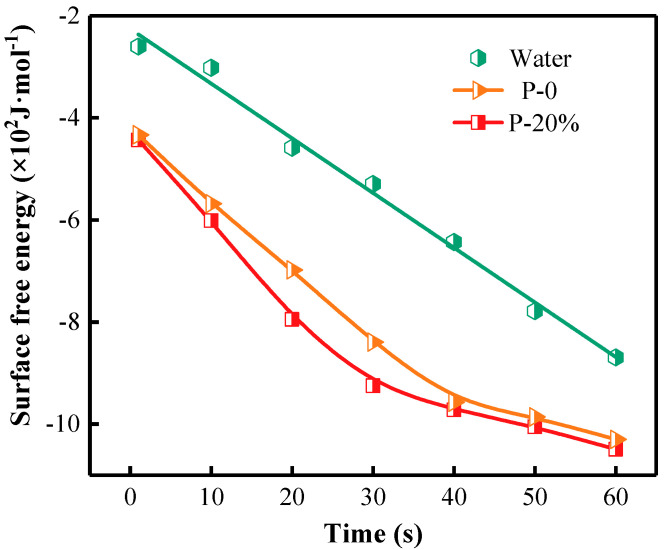
The surface-free energy on the wood surface due to water, P-0 and P-20% wetting.

The values of the surface-free energy of the wood surface wetted with water, P-0 and P-20% pre-gel droplets are shown in Figure 9. For the wood surface covered by water, the surface-free energy decreased linearly in time. In the cases of P-0 and P-20%, the surface- free energy decreased rapidly with the spread of the liquid droplets within the first 30 s and gradually within 30–60 s. The results indicate that the wettability of hydrogel droplets on the wood surface gradually increased and tended toward stability with the spread of the droplet. The surface-free energy for the systems with P-20% and P-0 decreased by 20.71% and 18.50% at 60 s compared with the wood–water pair. These results further demonstrated that water cannot easily spread on and wet the wood surface. P-20% has the best spreadability and wettability on the wood surface, most likely due to favorable contributions brought by both adequate viscosity and hydrophilicity of P-20% pre-gel mixture in a direct interaction with such a hydrophilic solid surface.

#### 2.4.2. Wettability of the Gels after the Phase Transition

**Figure 10:**
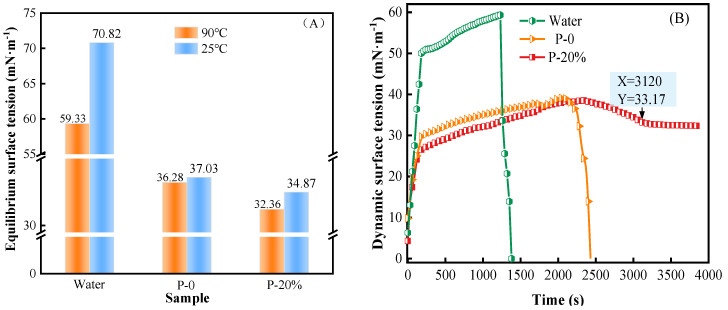
(**A**) Equilibrium surface tension of P-0 and P-20% at 90 °C; (**B**) Dynamic surface tension of P-0 and P-20% within 4000 s at 90 °C.

The equilibrium surface tension and the dynamic surface tension of water, P-0 and P-20% at 90 °C over a period of time of 4000 s are shown in Figure 10. 

As shown in Figure 10A, compared with water and P-0, the equilibrium surface tension of P-20% gel after phase transition decreased by 45.50% and 10.80%, respectively. This aspect could be due to a large content of surface-active components (HPMC, PEG, CS) in the P-20% hydrogels compared to water solely, with a tendency of increasing as a result of water evaporation. On the other hand, the presence of acetate anion as chemical species also endowed with surface-active character led to a lower surface tension for P-20% by comparison with that for P-0, which further improved the wettability of P-20% gel.

Due to the harsh conditions for surface tension measurements (900 °C, convection effect, water evaporation), it was quite difficult to reach the equilibrium values for the systems investigated (Figure 10B). As expected, the values of surface tension for the P-20% gel are well below those of water over the entire common period of time. The values a little lower than those of the P-0 gel were obtained for the P-20% system over a much larger common period of time while the possibility of continuing measurements in time could be extended up to about 4000 s for the P-20% system. This last feature mirrors a great capacity of water retention in the P-20% mixture over a long period of time that makes this porous gel the best system when it comes to its wettability and adhesiveness towards the chosen wood surface.

## 3. Conclusions

The wettability of HPC thermosensitive porous systems with different concentrations in water was investigated systematically. The HPC porous hydrogel concentrate was successfully prepared by physical cross-linking, and the phase transition occurred in only 28 s at 67 °C. The HPC porous systems with a concentration greater than 15 wt% exhibited an almost intact wet gel film at 200 °C. The water retention and thermal stability of gels were significantly improved given that the porous structure P-20% has the best spreadability and wettability on the wood surface, most likely due to favorable contributions brought by both adequate viscosity and hydrophilicity of P-20% pre-gel mixture in a direct interaction with such a hydrophilic solid surface. The values of surface tension for the P-20% gel after phase transition are well below those of water over the entire common period of time. This porous gel has a great capacity of water retention over a long period of time. The adhesion ratio of the gel increased by 32.67%, and the equilibrium surface tension decreased by 45.50%. It was the best system when it comes to its wettability and adhesiveness towards the chosen wood surface.

## 4. Materials and Methods

### 4.1. Materials

HPMC (methoxy = 28%–30%; specifications = 30 mPa·s; powder) was provided by Shanghai Yien Chemical Technology Co., Ltd. (Shanghai, China). CS (deacetylated degree > 90%; MW = 20–40 kDa; degree of deacetylation > 90%) was obtained from Shandong Youso Chemical Technology Co., Ltd. (Shandong, China). PEG (MW = 6 kDa) was supplied by Sinopharm Group Chemical Reagent Co., Ltd. (Shanghai, China). Na_2_CO_3_ and CH_3_COOH (36% concentration of its aqueous solution) were purchased from Tianjin Damao Chemical Reagent Factory (Tianjin, China).

### 4.2. Synthesis of HPMC/PEG/CS Thermosensitive Porous Hydrogel Concentrate

**Figure 11:**
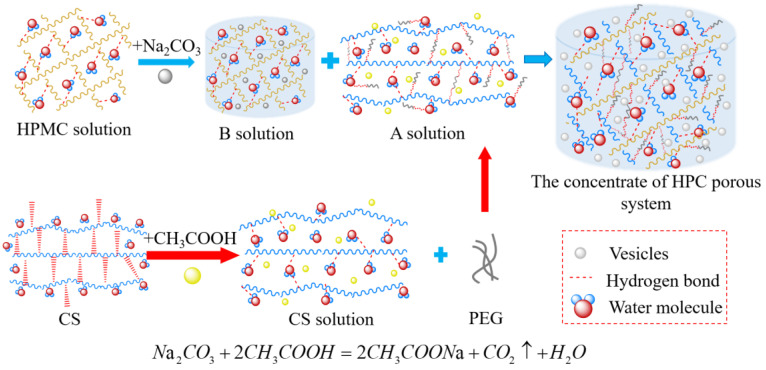
The synthesis process of the HPC porous concentrate.

The synthesis process of HPC porous hydrogels is outlined in Figure 11. CH_3_COOH solution was diluted to 0.375 wt%. CS was dissolved in the diluted CH_3_COOH solution and stirred at room temperature for 1 h to form the 0.4 wt% CS solution. PEG (1.5 wt%) was added to the CS solution and stirred until it was completely clear to obtain solution A. Na_2_CO_3_ (0.3 wt%) was poured into 3.0 wt% HPMC solution and stirred at room temperature for 2 h to obtain solution B. The components and concentration of HPC porous hydrogel are shown as Table 3.

Solution B was slowly poured into solution A and stirred for 3 h. CH_3_COOH reacted with Na_2_CO_3_ to produce CO_2_, which further generated vesicles uniformly dispersed throughout the final system (called HPC concentrate). The concentrate of the HPC porous system was then generated. At the same time, the HPC system mixture without the foaming agent (Na_2_CO_3_/CH_3_COOH) was also prepared.

### 4.3. Equipments and Procedures

#### 4.3.1. Testing the Basic Properties of the HPC Porous Systems

An infrared spectrometer (NICOLETIN10 instrument, Semmerfeld Technology Co., Ltd. (Saskatoon, SK, Canada)) was used to acquire IR spectra. (Every single IR spectrum was recorded as an average of 32 scans in the wavenumber range of 40–4000 cm^−1^) of the HPC porous system, HPC system, HPMC, PEG and CS.

The LCST and phase-transition response time of the HPC porous system concentrate was determined by tube inverting test.

A scanning electron microscope (FEI Quanta 450 FEG instrument, Semmerfeld Technology Co., Ltd.) was employed to investigate the morphology of the pre-gels and the gels framework after phase transition of the HPC porous and HPC systems. The pre-gels were freeze-dried in a lyophilizer (−58 °C, 0.22 mbar) for 24 h. The gels were dried at 90 °C for 24 h while the cross-section of the samples were sprayed with gold. In addition, the macro-morphology of the HPC porous hydrogels with different concentrations at 100 °C, 150 °C and 200 °C was observed.

The values of dynamic surface tension for water, P-0 and the HPC porous aqueous pre-gel with different concentrations within 500 s at 25 °C were determined using an automatic surface tensiometer (QBZY-3 instrument, Shanghai Fangrui Instrument Co., Ltd. (Shanghai, China) accuracy to within 0.001 mN·m^−1^). The dynamic surface measurements for P-20%, P-0 and water over a time period of 4000 s at 90 °C were measured. All the tests were repeated at least three times to obtain an average.

The values of contact angle for water, P-0 and the HPC porous aqueous pre-gel with different concentrations within 60 s on the batten surface were measured by a contact angle tester (JC2000D instrument, Shanghai Zhongchen Digital Technology Equipment Co., Ltd. (Shanghai, China)). The contact angle was calculated according to the Ɵ/2 method by using CAST3.0 software. The resolution of the contact angle could reach 0.01°. All the tests were repeated at least three times to obtain an average.

#### 4.3.2. Adhesion Weight Ratio Measurements

According to a number of preliminary tests, the surface of the wood batten used (35 mm × 35 mm × 150 mm) could be fully covered by using an amount of 30 g P-20.

The procedure was followed using water, P-0 and P-20% spread onto the batten surface. The total weight of the covered batten was determined after 300 s and 600 s at 900 °C (to fall beyond the LCST for the P-0 and P-20% systems and to avoid gel-to-sol transition), and the quantity adhesion weight ratio (°C) was calculated according to Equation (5).

(5) $$k=\frac{m_{t}-m_{0}}{30}\times 100\%$$

where *m*_0_ (in g) is the mass of the original batten.

## Figures and Tables

**Table 1:** Formulations of the HPC porous hydrogels with different water content.

Samples	HPC Porous Systems (wt%)	HPC Systems (wt%)	Water (wt%)
P-0	0	20	80
P-5%	5	0	95
P-10%	10	0	90
P-15%	15	0	85
P-20%	20	0	80
P-25%	25	0	75

**Table 2:** The adhesion weight ratio of water, P-0 and P-20% in the gel state.

Samples	300 s		600 s	
	Horizontal Batten	Vertical Batten	Horizontal Batten	Vertical Batten
Water	3.43%	1.30%	3.20%	1.20%
P-0	36.05%	29.05%	28.93%	24.95%
P-20%	41.23%	37.11%	35.87%	30.04%

**Table 3:** The components and concentration of HPC porous hydrogel.

Component	HPMC (wt%)	PEG (wt%)	CS (wt%)	CH_3_COOH (wt%)	Na_2_CO_3_ (wt%)
Concentration	3.0	1.5	0.4	0.375	0.3

## Data Availability

Data are contained within the article.
